# How to stimulate employees’ innovative behavior: Internal social capital, workplace friendship and innovative identity

**DOI:** 10.3389/fpsyg.2022.1000332

**Published:** 2022-09-20

**Authors:** Xiaoyang Zhao, Changjun Yi, Chusheng Chen

**Affiliations:** School of Business Administration, Huaqiao University, Quanzhou, China

**Keywords:** internal social capital, innovative identity, workplace friendship, employees’ innovative behavior, psychological factors

## Abstract

With the digital transformation of the economy and the rise of community innovation, how stimulating employees’ innovative behavior (EIB) becomes the basis for building sustainable competitive advantage in organizations. However, research has yet to systematically investigate the effect of internal social capital (ISC) on EIB. Based on social identity theory and resource conservation theory, this paper constructs a model to explain the mediating role of II between ISC and EIB and the moderating role of workplace friendship (WF). Using SPSS 27 and Amos 24 to analyze the data of 284 questionnaires, the results show that (1) ISC has a positive effect on EIB, (2) II plays a partial mediating effect in the relationship between ISC and EIB, and (3) WF has a positive moderating effect on the relationship between ISC and EIB. The conclusion provides management insight and practical guidance for creating an internal organizational climate to promote EIBs.

## Introduction

In the current context, China’s economy is in a critical period of optimizing the industrial structure and transforming economic growth momentum. Leading high-quality development with scientific and technological innovation has become the primary task to grow the real economy. For enterprises, employees’ innovation is the foundation of organizational innovation, which reflects that an individual’s independent innovative behavior will directly affect corporate performance. Therefore, how to effectively improve employees’ innovative behavior (EIB) has become a key topic in management. EIB is that employees integrate their new ideas and new technologies into their daily work, which has a positive effect on the sustainable development of the organization and the realization of self-worth. Existing studies on EIB mainly focus on employees’ personality characteristics (Wu et al., 2014; McCormick et al., 2019; He et al., 2020; Jonsson and Kahler, 2022), organizational incentives method (Zhang et al., 2015; Li-Ying et al., 2016; Lombardi et al., 2020), organizational innovation atmosphere (Wang et al., 2017; Li et al., 2019; Yang and Qin, 2022) and organizational leadership style (Reade and Lee, 2016; Tian and Zhang, 2020; Akbari et al., 2021; Shin et al., 2022). However, with the digital transformation of enterprises and the rise of community innovation, the work forms, identities, and roles of employees have changed dramatically, with innovation roles becoming more prominent, work forms becoming more networked, and younger work teams focusing more on creating a free and harmonious working environment, thus giving employees a greater sense of identity. Innovative identity means that employees identify themselves as having a sense of responsibility for innovation in their work practices, and have the responsibility and obligation to try to introduce new technologies, procedures, or knowledge into their work. This raises a practical question for managers: what kind of working environment is conducive to shaping employees’ innovative identity and how to stimulate EIB?

Social capital is a social network resource embedded in interpersonal communication and organizational teams, which is precisely an important factor affecting the behavior of innovative team members. The theoretical framework of social capital includes three dimensions: structural, relational, and cognitive (Nahapiet and Ghoshal, 1998). The structural dimension refers to the overall relationship pattern among employees, including the density, connectivity, and hierarchy of interpersonal networks. The relational dimension reflects the trust, norms, identity, obligations and expectations formed by the interaction among employees. The cognitive dimension emphasizes the common coding, language and experience among employees. Existing research has begun to pay attention to the role of social capital in organizational innovation. Some scholars have pointed out that internal social capital (ISC), such as internal cooperation, employees’ common experiences, employees’ interactive identity, etc., has a positive impact on organizational innovation (Campbell, 2020; Zhou et al., 2021), and ISC affects EIB through relational psychological contracts, knowledge sharing, and dual learning (Ng et al., 2010; Dong et al., 2017; Cohen and Ehrlich, 2019; Kiazad et al., 2019; Li H. H. et al., 2021; Ye et al., 2021; Zhang et al., 2022). Although the influence of ISC on innovation behavior has been examined, the discussion on the relationship mechanism between the two has yet to be deeply explored, and the influence mechanism between ISC, innovative identity, and innovative behavior needs to be further studied.

In addition, the flat and networked organizational structure is more and more widely recognized in the digital age. The relationship between employees is becoming more and more equal, thus the organizational atmosphere changes and the possibility of forming friendships in the team increases (Tsang et al., 2012; Goh et al., 2014), which also shows that workplace friendship (WF) plays an important role in modern organizational management. WF is an informal relationship formed and maintained between employees to bring mutual spiritual friendship needs (Rumens, 2017), which has significant effects on engagement (Yan et al., 2021), task performance (Methot et al., 2016), and innovation performance (Kuo et al., 2014; Abdulmuhsin and Tarhini, 2022). However, the existing literature does not directly answer how WF affects the relationship between ISC and EIB. Based on the resource dependence theory, the accumulation of employees’ ISC directly affects EIB. WF, as an informal interpersonal relationship, fits the individual needs of modern employees, which can make up for the resource consumption of EIB. Therefore, it is necessary to explore the heterogeneous impact of WF on the relationship between ISC and EIB. Then, can different degrees of WF play a moderating role in the relationship between employees’ ISC and innovative behavior? An in-depth investigation of this question can not only expand the relevant literature on the informal relationships in the context of community innovation but also help to further explore the conditional boundary of the impact of ISC on EIB. Based on resource dependence theory and social identity theory, this study takes employees’ innovative identity as a mediator variable to explore the effect of ISC on EIB and analyzes the moderating effect of WF on the relationship between ISC and EIB, which reveals the internal mechanism of EIB and finds the path and boundary to promote EIB.

This study provides research contributions in several primary ways. Firstly, this study extends prior research on the connection between ISC and EIB. Existing research mainly focuses on the relationship between social capital and organizational innovation (Yang et al., 2014; Chen et al., 2016), and lacks research on the impact of ISC on employee innovation behavior at the individual level. Secondly, some scholars have paid attention to the influence of ISC on EIB (Cohen and Ehrlich, 2019; Li Y. B. et al., 2021; Zhang et al., 2022), but they have not discussed the influence mechanism of behavior from the perspective of identity. This study introduces the variable of innovation identity into the research framework of social capital and employee innovation behavior. We focus on the mediating effect of innovative identity, which provides a certain reference value for the application of cognitive-behavioral research in organizational management. Thirdly, this study further discusses the moderating effect of WF in the relationship between ISC and EIB. This research can not only analyze the influence boundary of ISC on EIB but also provide certain policy suggestions for organizational teams to motivate EIB.

The remainder of this paper proceeds as follows. The section following builds the theoretical model, employing prior literature on the sources of EIB, ISC, WF, and innovative identity. Subsequently, the research methodology, data collection procedures, variables measurement, and the respondent sample are described. Results of construct validation and model testing employing the structural equation model are then reported. The paper concludes with a summary of study findings, highlighting contributions, implications, limitations and directions for future research.

## Literature review and hypotheses

### Internal social capital and employees’ innovative behavior

The resource dependence theory points out that the resources owned by employees in a complex and changeable work environment are key elements in achieving high-quality work performance. Individual employees build social networks with internal and external organizations and thus obtain the resources they need to operate and innovate from team members or the external environment. This particular resource possessed by employees is valuable and affects the behavior of individuals and groups. Individual social capital is mainly concentrated on the sum of resources at the individual level that helps to achieve their own value goals. If employees have more external social capital, they can influence organizational or team activities by sharing information and technical collaboration. This study mainly focuses on the ISC at the employee level, specifically the relational resources between employees and other members of the team. So, how does ISC affect EIB? Scholars have mainly argued the relationship between the two from the connotation of ISC. First, organizational information capital improves employees’ ability to acquire and absorb new technologies and knowledge (Ullah et al., 2021). When the social network among organizational members is closer, the frequency of information exchange among them will increase with the enhancement of interaction. The rich internal information capital has become an important source channel for employees to obtain industry and market information, which enables employees to better understand the future development prospects and direction of the organization (Sozbilir, 2018), thereby increasing the innovation opportunities and stimulating the innovative behaviors of employees. Secondly, the position of employees in the organizational network affects EIB. Diverse network lines reduce the cost of employees’ knowledge search and integration. For example, an employee located at the center of the network has more relationships, contributing to the interdependence and interaction with other employees directly connected to him/her, which has a positive effect on the creation of an organizational innovation climate (Kumar et al., 2022). Finally, abundant ISC helps to form a good innovation environment, which helps to stimulate the creative thinking of employees. Individual employees generate creative thinking in their interactions with other team members, rather than relying solely on their abilities and actions (Lei et al., 2021), which also reflects that a good team relationship is conducive to the formation of an innovative atmosphere. When an employee is located at the center of a social network, the employee has more access to resources than other employees. Abundant social capital can promote employees’ knowledge sharing and the development of organizational innovation activities, which shows that knowledge sharing within the organization directly affects EIB (Hu and Randel, 2014; Obrenovic et al., 2020; Halisah et al., 2021). In the digital age, the widespread application of work communities within organizations helps to link employees and better share network resources. Abundant ISC cannot only enable employees to communicate and cooperate more widely in diverse team relationships but also help to improve employees’ absorptive capacity and stimulate EIB. Hence, this paper proposes:

Hypothesis 1: ISC has a positive impact on EIB.

### The mediating role of innovative identity

EIB is not only a decision-making behavior of employees but also a social behavior made by employees based on their value orientation (Luoh et al., 2014; Nazir et al., 2021). This kind of social behavior can differ depending on the values (Purc and Laguna, 2019), which indicates that employees’ self-innovative identity is an important factor affecting the innovation of enterprises. Social identity theory suggests that individuals’ behavioral decisions are largely influenced by their social identity and that there is a high degree of consistency between the social values and emotions of individuals and their social identity. The social capital (relationship) of employees we mentioned above is precisely an important factor affecting social identity. The basic premise of employees’ identity is that people have information about the category to which they belong, including the individual’s knowledge about one’s belonging to a certain social group and the emotional and value meaning of their group membership. Employees in an organizational team have similar basic categories of information such as work environment, language, and cultural atmosphere that form a certain identity. Personal identity within an organization reflects the degree to which individuals identify themselves as “insiders” within the group in a specific context (He et al., 2014; Liu et al., 2020), and this sense of “insiders” identity is precisely derived from the differences between individuals’ self-evaluation classification and comparison with groups. Employees with a high sense of identity emphasize “group” and “identity responsibility,” and engage in innovative behaviors that are conducive to the development of organizational operations (Chen et al., 2021). In an employee’s social relationship network, each employee with a unique category persists in the relational structure as part of a group attribute, and this social identity description defines the attributes of an individual as a group member. Therefore, the innovative identity attributes of employees in innovation teams will be more pronounced. Existing literature shows that employees with a high sense of identity have higher levels of engagement (Wang et al., 2020; Li H. H. et al., 2021), job satisfaction (Al-Ghazali et al., 2021; Krug et al., 2021), and innovation (Tang and Naumann, 2016). From a theoretical perspective, employees’ ISC can influence their behavioral decisions by changing how they perceive in the team. Trust is an important dimension of social capital, a prerequisite for focal companies or employees to conduct extensive innovation searches in diverse network relationships. Therefore, the high level of trust brought by rich social capital leads to more open and inclusive cooperation among members in the organization, and strengthens the sense of their innovative identity, which ultimately motivates EIB. Employees with rich ISC are more likely to stimulate their innovative thinking in the process of interaction and communication. The co-working environment, language, and cultural atmosphere make employees aware of their innovative role in the organization, thus strengthening their motivation and willingness to bring new ideas and technologies to the team. Accordingly, this paper proposes:

Hypothesis 2: II plays a mediating role in the relationship between ISC and EIB.

### The moderating role of workplace friendship

WF is an informal institution that reflects the degree of employee relationships with other members of an organizational team. WF is a special relationship that not only serves individual or organizational goals but also provides employees emotional support. Nielsen et al. (2000) divided WF into two dimensions, opportunity and intensity, where friendship opportunity refers to allowing an informal relationship between employees of an enterprise, while friendship intensity represents the degree of closeness between the two parties who established an informal relationship. There is a substantial body of research on the influencing factors and economic consequences of WF. The heterogeneity of work situations is an important factor affecting WF, which specifically involves leadership styles (Kohan et al., 2018), organizational culture and other aspects (Ozbek, 2018). Workplace friendship can stimulate communication and voice behavior among team members (Lee et al., 2022), and positively promote unethical pro-organizational behavior through emotional commitment. In addition to creating a good cultural atmosphere and enhancing feelings among colleagues, WF also plays an important role in absorbing and integrating key resources for employees (Mao, 2022). Resource conservation theory shows that organizational members maintain their resource levels primarily through resource acquisition and loss avoidance. EIB is an extremely time and energy-consuming activity, which makes employees less motivated to innovate in the face of a lack of resources. However, good WF leads to close relationships and frequent communication among organizational members, which can improve employees’ social capital. At the same time, WF also promotes the level of emotional support, technical support and information support in employees’ social networks. This means that employees have access to more key resources through WF to compensate for their loss of resources to carry out innovative activities. All human activities are rational behaviors in pursuit of self-interest, as a result, employees have greater opportunities to communicate in high WF situations. The accumulation of ISC of employees increases continuously as their emotions are satisfied. In this case, employees have a more innovative sense of responsibility and team ownership, which leads to individual behaviors that benefit the organization. Therefore, the innovation cost of employees will decrease with the enrichment of ISC under high WF, which makes their innovation motivation stronger. Conversely, in an environment with low WF, although greater social capital can broaden employees’ access to key resources, employees still worry about facing unfriendly behavior such as gossip or hit from colleagues when their innovation activities fail. Therefore, employees in environments with low WF are likely to choose to reduce innovative behaviors to maintain existing resources and reduce losses. To sum up, this paper proposes:

Hypothesis 3: WF has a positive moderating effect on the relationship between ISC and EIB.

**FIGURE 1:**
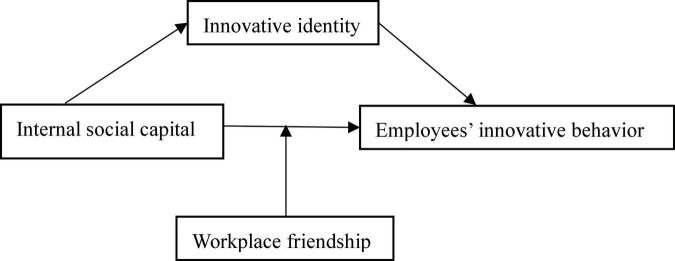
A research framework.

Based on the above analysis, this study presents the research framework among ISC, innovative identity, WF, and EIB in Figure 1.

## Research methodology

### Sample description

This research uses a questionnaire survey to collect data and selects enterprises in Beijing, Shanghai, Guangzhou, Chengdu, Dongguan, Taiyuan, Xiamen, and other places as the research objects, involving employees in manufacturing, Internet, finance, and education industries. The scales used in this study are based on maturity scales in previous research. In the process of designing the items of the scale, we invite three professors in the same field to translate and back-translate the scales, and finally the scales are modified and improved for the context of this study. The main contents of the questionnaire include employee-level ISC, innovative identity, WF, and innovative behavior. All items are expressed on a five-point Likert scale (where “1” means totally disagree and “5” means totally agree). We distribute a total of 387 online questionnaires. After excluding invalid questionnaires with answer time less than 200 s, education below high school, and excessively regular or missing answers, we finally get 284 valid questionnaires, with an effective rate of 73.4%.

### Variables

#### Independent variable

##### Internal social capital

ISC this study mainly examines the relational dimension of ISC. A high level of trust among team members increases their closeness and collaboration, which fosters a shared learning atmosphere within the organization. Therefore, according to the research of Tsai and Ghoshal (1998), we mainly include 4 items in the scale such as “My relationships with colleagues are more important than my achievements,” “I can fully rely on the ability of my colleagues to work,” “It is very convenient for me to share information with my colleagues,” “My colleagues and I share common development goals.” The Cronbach’s alpha of this scale is 0.873, which has a high-reliability level.

#### Dependent variable

##### Employees’ innovative behavior

EIB innovative behavior means that employees incorporate new ideas, new ideas, and new procedures into their daily work. This innovative behavior has a positive impact on organizational productivity and team management efficiency. According to the research of Scott and Bruce (1994), we mainly include 4 items in the scale such as “I will explore new technologies, new processes, skills or new products at work,” “I often have some creative ideas or ideas at work,” “I come up with innovative ideas at work and seek support from colleagues,” “I seek funding and resources to implement innovative ideas at work.” The Cronbach’s alpha of this scale is 0.888, which has a high-reliability level.

#### Mediator variable

##### Innovative identity

II mainly refers to employees’ recognition that they should have a sense of responsibility for innovation in their work practices, and have the responsibility and obligation to try to introduce new technologies, procedures or knowledge into their work. Following Smidts et al. (2001) and Song et al. (2018), the scale mainly includes 3 items such as “I feel that I have an obligation to try to introduce new procedures into my work,” “I consider myself responsible for bringing about change at work,” “I have a responsibility to correct problems in the organization.” The Cronbach’s alpha of this scale is 0.835, which has a high-reliability level.

#### Moderator variable

##### Workplace friendship

This study mainly examines the intensity dimension of WF. Referring to the research of Li G. H. et al. (2021), we include 3 items in the scale such as “My colleagues are not trustworthy, so I usually don’t accept their help at work,” “I have developed strong friendships with colleagues at work,” “I had the opportunity to get to know my colleagues.” The Cronbach’s alpha of this scale is 0.814, which has a high-reliability level.

Besides, we take individual gender, age, organizational level, educational level, and years working as control variables.

### Sample distribution

In this part we analyze the demographic variables. The final sample has 284 valid observations., in which 137 (48.24%) are males and 147 (51.76%) are females. Most of the employees in the sample are between the ages of 20 and 39, of which 27% are 20–29 years old, and 60.21% are 30–39 years old. In terms of organizational level, the proportion of grassroots employees, grassroots managers, middle managers, and top managers are 33.45%, 29.93%, 26.76% and 9.86%, respectively. As for educational background, 3.87% of the sample are high school or technical secondary school, 8.80% are junior college, 67.61% are undergraduate, and 19.72% are master’s degree or above. The working years of employees are mostly distributed between 1 and 10 years, of which 9.51% of employees have worked for 5 years or less, 82.39% for 6–10 years, 4.58% for 11–15 years, and 3.52% for 16 or more than. The specific demographic characteristics are described in Table 1.

**TABLE 1:** Sample distribution of demographic characteristics.

Variables	Category	Frequency	Percentage
Gender	Male	137	48.24%
	Female	147	51.76%
Age	20-29	77	27.11%
	30-39	171	60.21%
	40-49	19	6.70%
	50-59	16	5.63%
	60 years old and above	1	3.5%
Organizational level	Entry level employee	95	33.45%
	Grassroots managers	85	29.93%
	Middle management	76	26.76%
	Senior management	28	9.86%
Educational level	High school or technical secondary school	11	3.87%
	Junior college	25	8.80%
	Undergraduate	192	67.61%
	Master degree and above	56	19.72%
Years working	5 years and below	27	9.51%
	6-10	234	82.39%
	11-15	13	4.58%
	16-20	4	1.41%
	21 years and above	6	2.11%

### Reliability and validity analysis

Exploratory factor analysis is performed on the data using SPSS 21.0, and the reliability analysis results of the variables are shown in Table 2. In terms of structural validity, the factor loadings of all items that constitute ISC, EIB, innovative identity, and WF are all greater than 0.5. The Cronbach’s alpha values of all variables also meet the criterion of greater than 0.65 (the Cronbach’s alpha values of ISC, EIB, innovative identity and WF are: 0.812, 0.813, 0.721, 0.675, respectively), indicating good reliability and internal consistency of each variable measure. In addition, the mean value of average variance extracted (AVE) is also greater than 0.7, which meets the discriminant criteria. The combined reliability Composite reliability ratings (CR) values of ISC, EIB, innovative identity, and WF are 0.869, 0.886, 0.756, and 0.890, all of which are greater than 0.7, indicating high reliability.

**TABLE 2:** Exploratory factor analysis.

Constructs	Items	Factor loadings	Cronbach’ alpha	CR	AVE
Internal social capital	ISC1	0.719	0.812	0.869	0.791
	ISC2	0.807			
	ISC3	0.820			
	ISC4	0.811			
Employees’ innovative behavior	EIB1	0.797	0.813	0.886	0.813
	EIB2	0.817			
	EIB3	0.828			
	EIB4	0.809			
Innovative identity	II1	0.753	0.721	0.756	0.715
	II2	0.754			
	II3	0.626			
Workplace friendship	WF1	0.875	0.675	0.890	0.850
	WF2	0.899			
	WF3	0.782			

Factor analysis is performed using AMOS21.0, from which the discriminant validity can be obtained and the results are shown in Table 3. In this table we can see that the fit of the four-factor model to the actual observed data (χ^2^/df = 1.516; GFI = 0.951; NFI = 0.954; CFI = 0.984; RMSEA = 0.043) is significantly better than the alternative models of three-factor, two-factor, and one-factor, which indicates that the four-variables involved in this study has good discriminant validity.

**TABLE 3:** Confirmatory factor analysis.

Model	χ*^2^*	*df*	χ*^2^*/*df*	GFI	NFI	CFI	RMSEA
Four-factor	107.664	71	1.516	0.951	0.954	0.984	0.043
three-factor	432.723	74	5.848	0.828	0.816	0.841	0.131
Two-factor	660.851	76	8.695	0.729	0.718	0.741	0.165
Single factor	909.820	77	11.816	0.643	0.612	0.631	0.195

Four-factor model, internal social capital, innovative identity, workplace friendship and innovative behavior; Three-factor model, internal social capital, innovative identity + workplace friendship, innovative behavior; Two-factor model, internal social capital + innovative identity, Workplace friendship + innovative behavior; Single factor model, internal social capital + innovative identity + workplace friendship + innovative behavior. “ + “ means mixed.

## Empirical analysis

### Common method variation test

Firstly, in order to reduce the common method bias in the study, we adopt a reverse scoring for some items in the research design. Secondly, we analyze the degree of common method variation (CMV) using Harman’s one-way test. The results show that the factor one explains 23.43%, which is less than the 50% criterion. Factor one does not explain most of the variance, which indicates that there is no homoscedasticity problem. Finally, this study adds common method factors for further testing. The comparison results with the four-factor model are shown in Table 4, from which we can see that the increase of GFI, NFI, and CFI after adding the common method factor does not exceed 0.1, and the decrease of RMSEA does not exceed 0.05, In addition, the results of comparing the χ^2^ and df of the two models are as follows:Δχ*^2^* = 802.156, Δdf = 6, *P* < 0.001, thus it can be concluded that there is no obvious common method bias in this study.

**TABLE 4:** Common method bias test.

Model	χ*^2^*/*df*	GFI	NFI	CFI	RMSEA
Four-factor	1.516	0.951	0.954	0.984	0.043
Add common method factor	1.406	0.963	0.966	0.990	0.038

### Descriptive statistical analysis and correlation analysis

The mean, standard deviation and correlation coefficient of each study variable are shown in Table 5, in which there is a significant positive correlation between ISC and EIB (*r* = 0.475, *p* < 0.01), is a significant positive correlation between ISC and innovative identity (*r* = 0.582, *p* < 0.01), and a significant positive correlation between innovative identity and employee innovation behavior (*r* = 0.682, *p* < 0.01), while there is no significant relationship between WF and ISC and EIB.

**TABLE 5:** Descriptive statistics and correlation analysis.

Variables	Mean	*SD*	1	2	3	4	5	6	7	8	9
1.Gender	1.518	0.501	1.000								
2.Age	2.919	0.768	–0.038	1.000							
3.Educational level	4.032	0.664	0.036	0.188***	1.000						
4.Years working	2.042	0.622	0.009	0.376***	–0.029	1.000					
5.Organizational level	2.130	0.991	–0.087	0.384***	0.047	0.146**	1.000				
6.Internal social capital	3.690	0.784	–0.067	0.189***	0.140**	0.078	0.072	1.000			
7.Innovative identity	3.948	0.736	0.044	0.261***	0.168***	0.064	0.143**	0.582***	1.000		
8.Workplace friendship	3.330	0.967	0.055	–0.078	–0.058	0.132	0.030	0.089	0.051	1.000	
9.Employees’ innovative behavior	3.777	0.792	–0.009	0.247***	–0.094	0.059	0.215***	0.475***	0.682***	0.029	1.000

***p < 0.01, **p < 0.05.

### Empirical results analysis

We conduct an empirical analysis on Hypothesis 1, and the regression results are shown in Table 6. Model 2 shows that the coefficient of ISC is 0.453 and significant at the 1% level, which indicates that ISC has a positive effect on EIB. This finding suggests the rich ISC provides employees with extensive opportunities for communication and cooperation, which helps to improve employees’ knowledge absorptive capacity and acceptance of new knowledge. Therefore, Hypothesis 1 is supported.

**TABLE 6:** Benchmark regression analysis results.

Variable type	Variables	Innovative behavior	Innovative behavior
		Model 1	Model 2
Control variables	Gender	0.020	0.059
	Age	0.197***	0.136**
	Educational level	–0.080	–0.021
	Years working	–0.008	–0.015
	Organizational level	0.120**	0.116**
Independent variable	Internal social capital		0.453***
	*R* ^2^	0.084	0.274
	*F*	5.075***	17.179***

***p < 0.01, **p < 0.05.

### Mediating effect analysis

We conduct regression analysis according to the commonly used mediation test steps, and the results are shown in Table 7. Firstly, Model 3 shows that ISC has a significant positive impact on innovative identity (β = 0.521, *p* < 0.001). Secondly, Model 4 shows that innovative identity has a significant positive effect on innovative behavior (β = 0.712, *p* < 0.01). Finally, take EIB as the dependent variable, we incorporate both ISC and innovative identity into the empirical model, and the results show that innovative identity has a significant positive impact on EIB (β = 0.638, *p* < 0.01). At the same time, ISC still has a significant positive impact on EIB, but the predictive effect is significantly reduced (β = 0.121, *p* < 0.01). Therefore, innovative identity plays a partial mediating role in the influence path of ISC on EIB. Our empirical regression results support Hypothesis 2.

**TABLE 7:** Mediating effect analysis results.

Variable type	Variables	Innovative identity	Employees’ innovative behavior	Employees’ innovative behavior
		Model 3	Model 4	Model 5
Control variables	Gender	0.045	0.137*	–0.028
	Age	0.047*	0.140**	0.047
	Educational level	–0.025	–0.080	0.030
	Years working	–0.004	–0.013	–0.007
	Organizational level	0.08*	0.049	0.084**
Independent variable	Internal social capital	0.521***		0.121**
Mediating variable	Innovative identity		0.712***	0.638***
	*R* ^2^	0.274	0.382	0.491
	*F*	17.179***	28.557***	37.919***

***p < 0.01, **p < 0.05, *p < 0.1.

In addition, Table 8 shows that the upper and lower bounds of the Bootstrap 95% confidence interval of the direct effect of ISC on EIB and the mediating effect of innovative identity do not contain 0, indicating that ISC can not only directly affect EIB, but also have an impact on EIB through the mediating effect of innovative identity. The direct effect (0.121) and the mediating effect (0.332) account for 26.71 and 73.29% of the total effect (0.453).

**TABLE 8:** Decomposition table of the total effect, direct effect and mediating effect.

	Effect value	Boot	Boot LLCI	Boot ULCI	Relative effect value
Total effect	0.453	0.053	0.349	0.558	
Direct effect	0.121	0.054	0.014	0.228	26.71%
Mediation effect	0.332	0.051	0.235	0.453	73.29%

### Moderating effect analysis

The results of the moderating effect of WF between ISC and employee innovation behavior are shown in Table 9. It can be seen that the interaction item between ISC and WF has a significant positive impact on EIB (β = 0.136, *p* < 0.001), which indicates that with high WF, the effect of ISC promotes EIB is more pronounced. Therefore, Hypothesis 3 is supported.

**TABLE 9:** Moderating effect regression results.

Variable type	Variables	Innovative behavior	Innovative behavior
		Model 2	Model 6
Control variables	Gender	0.059	0.018
	Age	0.136**	0.121*
	Educational level	–0.021	–0.024
	Years working	–0.015	–0.016
	Organizational level	0.116**	0.125***
Independent variable	Internal social capital	0.453***	0.444***
Moderator variable	Workplace friendship		–0.016
Interaction variable	Internal social capital*workplace friendship		0.136***
	*R* ^2^	0.274	0.295
	*F*	17.179***	14.231***

***p < 0.01, **p < 0.05, *p < 0.1.

**FIGURE 2:**
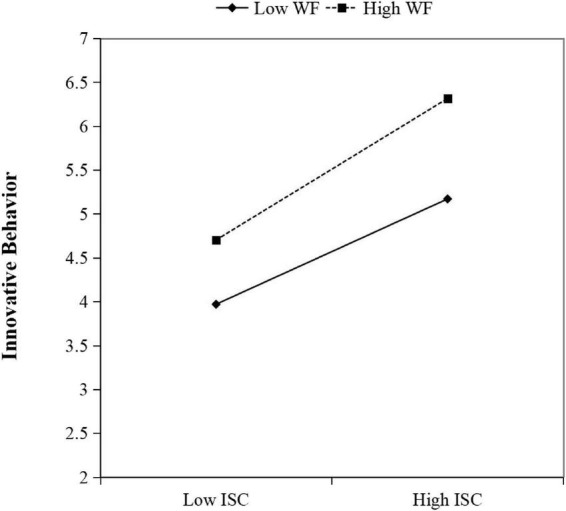
Moderating effect of workplace friendship.

Figure 2 presents a moderating effect diagram indicating the effect of WF on the relationship between ISC and EIB. The solid and dashed lines show a cross trend, which indicates that WF has a significant moderating effect. The solid line represents the influence of ISC on EIB under high WF. The dashed line represents the influence of ISC on EIB under low WF. Meanwhile, the slopes of the two lines in high WF and low WF are both positive, and the slope of EIB is greater in high WF group. This shows that employees with high WF have more innovative behaviors than those with low WF when their ISC increases. Therefore, the influence of ISC on EIB is stronger in the context of high WF.

## Discussion

### Conclusions

This study constructs a relationship model among ISC, innovative identity, WF, and EIB, and focuses on the mediating effect of employees’ innovative identity and the moderating effect of WF. Using structural equation model, multiple regression analysis and other methods to empirically test the collected data, this paper draws the following conclusions: (1) In this study, employees can learn new knowledge from other project groups through social network resources within the organization, which can improve employees’ innovation motivation and work performance. The above research conclusions are consistent with those of Reddy et al. (2021) and Zhang et al. (2022). (2) Existing research has confirmed the promotion effect of ISC on EIB (Dong et al., 2017; Kiazad et al., 2019; Zhang et al., 2022). This paper enriches the research on the impact of employee emotional factors on innovation Literature on behavioral influences. This study shows that abundant ISC promotes interaction and technical learning among team members, which enables employees to develop a sense of innovative identity. And this innovative identity has a positive effect on EIB, which means that innovative identity plays a mediating role in the relationship between ISC and EIB. (3) Then, our research shows that employees with high WF s will communicate with each other more frequently and get regular feedback about innovation from their workplace friends, which is consistent with the findings of Cao and Zhang (2020). This study further found that WF has a positive moderating effect on the relationship between ISC and EIB. In the context of higher WF, the positive effect of ISC on EIB is more significant.

### Theoretical contributions

EIB in the era of digital economy has become a hot research topic. Existing research mainly discusses the impact of external social capital on innovative behavior at the organizational level. However, the impact of ISC on EIB at the individual level is still controversial, and the impact mechanism is not yet clear. The theoretical contributions of this study are mainly in two aspects: Firstly, based on social identity theory and resource conservation theory, this paper embeds innovative identity into the relationship between ISC and EIB, and further discusses the role of innovative identity in the formation of EIB, which expands the perspective of EIB research, discovers the cognitive mechanism of the impact of ISC on EIB and responds to the call by related scholars that it is necessary to further explore the influence mechanism of ISC on EIB from the perspective of “identity” cognition. The conclusions of the study promote the cross-integration of sociology and management research. Secondly, in the context of Chinese culture, which emphasizes “interpersonal relationships,” this study explores the heterogeneous impact of WF on the relationship between ISC and EIB, and examines the boundary of the impact of ISC on EIB. From the emotional level with Chinese characteristics, this paper explains why the influence of ISC on EIB is different, which responds to the relevant scholars’ proposal to further analyze the reasons for the difference in the influence of ISC on employees’ behavior and provides theoretical support for how to create conditions to promote individual innovative behavior in management practice.

### Practical contributions

This study provides new insights and solutions for promoting the employees’ behavior. Innovation is the foundation for an enterprise to develop and maintain its competitive advantage. As an important resource in the modern workplace, the impact of ISC on EIB is an issue that scholars focus on. The samples of this survey are mainly employees born after 1985, who are usually considered to be the new generation of employees with unique personality and creativity. How to stimulate the innovative vitality of the new generation of employees to create value for the enterprise is an important practical problem faced by the enterprise. The conclusions of this paper have important practical value for guiding the innovative behavior of the new generation of employees. Firstly, the research results show that ISC can arouse the innovative identity of the new generation of employees, which can motivate their independent innovative behavior. With the development of the digital economy and dramatic changes in organizational structure, the new generation of employees is no longer subject to authority and shackles. Therefore, organizational leaders should pay attention to the importance of ISC, and create a good internal learning and communication platform for employees through quality development, team training and cross-departmental mutual aid groups, which can enhance the trust relationship and the ability to acquire social capital among employees. In addition, the new generation of employees pays more attention to their intrinsic value. Innovative sense of identity helps to improve work enthusiasm and thus generate innovative behaviors. Therefore, managers should take identity as one of the contents of daily management and performance assessment in management practice, and should strive to cultivate a culture of innovation and empower internal community innovation, which can enhance employees’ innovative identity.

Secondly, this paper shows that the relationship between ISC and EIB is affected by the level of WF. Under high WF, the innovation cost of employees will be significantly reduced with the increase of social capital, which will stimulate employees’ enthusiasm for innovation. However, under low WF, even though rich ISC can broaden the channels for employees to obtain key resources, innovative behavior is reduced due to unfriendly behavior among colleagues. As an informal interpersonal relationship, WF plays an important role in promoting the formation of EIB by ISC. Therefore, managers need to emphasize the trust and friendship among team members in addition to the positive role of ISC. In view of the new generation’s preference for freedom and equality, on the one hand, enterprises can create a comfortable workplace environment by establishing a “flat” organizational structure and adopting open office, group building and democratic management. On the other hand, enterprises can improve WF by organizational care and regular cross-departmental activities to meet the emotional needs of employees.

### Limitations and future research

This study also has some limitations. First, the items of the questionnaire are self-assessed by employees, and although there is no serious common method bias, it may still have an impact on the results. In the future, the article can adopt the mode of mutual evaluation and scoring between leaders and employees to reduce homologous variance. Second, this study mainly analyzes how to create an environment conducive to innovation within the organization. Future research can explore the role of personal factors such as self-motivation and efficacy identification on EIB, and further refine the moderating role of employees’ characteristic heterogeneity.

## Data availability statement

The raw data supporting the conclusions of this article will be made available by the authors, without undue reservation.

## Ethics statement

Written informed consent was obtained from the individual(s) for the publication of any potentially identifiable images or data included in this article.

## Author contributions

XZ and CY conceived and designed the experiments and collected and interpreted the data. CC analyzed the data, examined, critically contributed, and finally approved the manuscript. All authors contributed to the article and approved the submitted version.
